# Knowledge Graph-Based In-Context Learning for Advanced Fault Diagnosis in Sensor Networks

**DOI:** 10.3390/s24248086

**Published:** 2024-12-18

**Authors:** Xin Xie, Junbo Wang, Yu Han, Wenjuan Li

**Affiliations:** 1 School of Intelligent Systems Engineering, Sun Yat-sen University, Shenzhen 518107, China; xiex36@mail2.sysu.edu.cn (X.X.); wangjb33@mail.sysu.edu.cn (J.W.); 2 Department of Mathematics and Information Technology, The Education University of Hong Kong, Hong Kong SAR, China; wenjuan.li@my.cityu.edu.hk

**Keywords:** knowledge graph, in-context learning, large language models, fault diagnosis

## Abstract

This paper introduces a novel approach for enhancing fault diagnosis in industrial equipment systems through the application of sensor network-driven knowledge graph-based in-context learning (KG-ICL). By focusing on the critical role of sensor data in detecting and isolating faults, we construct a domain-specific knowledge graph (DSKG) that encapsulates expert knowledge relevant to industrial equipment. Utilizing a long-length entity similarity (LES) measure, we retrieve relevant information from the DSKG. Our method leverages large language models (LLMs) to conduct causal analysis on textual data related to equipment faults derived from sensor networks, thereby significantly enhancing the accuracy and efficiency of fault diagnosis. This paper details a series of experiments that validate the effectiveness of the KG-ICL method in accurately diagnosing fault causes and locations of industrial equipment systems. By leveraging LLMs and structured knowledge, our approach offers a robust tool for condition monitoring and fault management, thereby improving the reliability and efficiency of operations in industrial sectors.

## 1. Introduction

In the context of equipment maintenance, sensor networks are increasingly relied upon to provide critical data for detecting and diagnosing faults. The textual descriptions of faults derived from sensor data are essential for understanding the operational health of various industrial equipment. Ensuring the accuracy and reliability of fault diagnosis results derived from these data is crucial for maintaining operational integrity and efficiency. Traditional fault diagnosis methods, which often depend on static rules, are insufficient for addressing the dynamic and complex nature of equipment malfunctions. In recent years, the development of artificial intelligence, particularly the emergence of large language models (LLMs), has opened new avenues for interpreting sensor-derived text and enhancing fault diagnosis capabilities. For instance, prominent LLMs such as ChatGPT and ChatGLM have demonstrated exceptional proficiency in addressing various downstream tasks within a broad domain.

When interpreting sensor-derived textual descriptions of faults across various types of industrial equipment, LLMs may struggle to provide responses that exhibit the necessary domain expertise [1]. At times, their semantic capabilities fall short in generating answers that align with the specialized knowledge required for accurate fault diagnosis [2]. This deficiency in professionalism presents a significant challenge, as the responses generated by LLMs may lack the ability for precise maintenance and operational decisions. This poses significant challenges for text analysis and generation in specialized domains. Although fine-tuning pretrained models by adjusting parameters presents a potential solution, it becomes increasingly resource-intensive and time-consuming as model scales grow. To enhance the capabilities of LLMs for specialized tasks without the resource-intensive process of parameter updates, we turn to in-context learning (ICL).

ICL is particularly advantageous, as it can enhance the capabilities of LLMs for specialized tasks without parameter updates. By doing so, the excessive resource consumption issue associated with traditional fine-tuning methods is avoided. While employing LLMs for domain-specific text analysis, it often results in suboptimal performance without the guidance of task-related information. By using knowledge from a specific domain, a domain-specific knowledge graph (DSKG) [3] can be constructed for the analysis of domain-specific text. The DSKG provides a structured repository of knowledge that can be integrated with LLMs, thereby supplementing LLMs with high-quality demonstrations through ICL. This integration is expected to significantly improve the models’ performance in the specific domain. In this paper, we delve into a specific domain problem—the text-based analysis of causes behind wind turbine faults. We are motivated to investigate the applicability of ICL for domain-specific text analysis, questioning whether it can yield satisfactory results in this domain-specific analysis.

In this paper, we focus on the text-based analysis of causes behind equipment failures, investigating the applicability of ICL for such domain-specific text analysis. We introduce the KG-ICL method, a prompt-driven approach [4,5] that enhances the linguistic analytical capabilities of LLMs by incorporating structured knowledge from a DSKG tailored for equipment diagnostics [6]. Firstly, the approach begins with the creation of a DSKG, which serves as the foundation for retrieving specialized information. During the construction of the DSKG [7], a dilated gated convolutional neural network (DGCNN) is utilized to perform long-length entity and relationship extraction, particularly tailored to the unique characteristics of fault texts. Secondly, to retrieve task-relevant subgraphs from the DSKG, we propose the long-length entity similarity (LES) metric, which enhances the relevance of the retrieved knowledge. Thirdly, based on defined rules and domain-specific insights, prompts are generated for the specific domain, forming the basis on which LLMs provide feedback. Finally, we present various experiments applying the proposed KG-ICL method to fault cause analysis. We test three different prompt scenarios and nine different LLMs to validate the effectiveness of the task-relevant prompts generated by our method. The results demonstrate that with the KG-ICL method, LLMs across different parameter scales show improved performance, with accuracy increases ranging from 1% to 4.8%, confirming the effectiveness of our method.

The contributions of this article are as follows.

- We construct a unique fault text dataset that includes domain-specific expertise. This dataset includes approximately 1000 instances that encompass fault modes, causal analyses, and maintenance strategies. This dataset summarizes expert diagnostic knowledge, compensating for the lack of general availability of fault text datasets.
- We introduce KG-ICL, a method designed for fault cause analysis. We employ a DGCNN to construct a DSKG and use the LES metric to retrieve task-related knowledge. By means of predefined prompt templates, we obtain task-related demonstrations. Through KG-ICL, the structured knowledge contained in a knowledge graph (KG) can be provided to LLMs, offering them a referenceable background of domain-specific expertise for text analysis.
- We apply the KG-ICL method in a specific domain as an example, thereby validating the feasibility and soundness of the proposed approach in practical applications. Through a series of experiments, we systematically compare the performance of three types of prompts—without prompts, domain-relevant but not task-related prompts, and task-related prompts—using various LLMs.

The remainder of this article is organized as follows. Section 2 reviews related works on ICL, LLMs, and KGs. In Section 3, we detail the proposed KG-ICL method. Section 4 outlines our experimental settings and the results. In Section 5, we discuss and analyze the results of the experiments. Finally, Section 6 concludes this paper and highlights avenues for future research.

## 2. Related Work

### 2.1. LLMs and In-Context Learning

ICL [8] is distinguished by the fact that it does not involve attempting to modify model parameters but rather focuses on exploring methods that can effectively bring out the hidden capabilities of LLMs. The challenge here lies in designing appropriate prompts. In previous research, scholars have pursued two distinct types of prompt mechanisms: soft prompts and hard prompts [9,10]. Some approaches involve the generation of hard prompts based on predefined rules [11], while others use generative models to create prompts [12] that are more finely tuned. The main limitation of hard prompts is their potential to stifle a model’s creativity, leading to less diverse generated text. Nevertheless, in specialized domains, the need for diversity in the generated results is not particularly emphasized. In contrast, continuous prompts (or soft prompts) no longer involve specific tokens but rather employ pseudotokens [13,14,15], which lack inherent meaning. Accordingly, such parameterized continuous prompts may exhibit limitations in terms of both interpretability and readability.

The design of the verbalizer also significantly influences the performance of an LLM. With the introduction of a KG, it becomes feasible to bypass manual selection of label words through the integration of external knowledge [16]. However, the means of introducing structured knowledge from a KG during prompt generation has yet to be explored.

Regarding on how ICL functions in the prediction process, Min et al. [17] posited that LLMs acquire patterns of label distribution and data organization from demonstrations. This occurs independently of the correlation between sample prompts and label words. However, in subsequent research by Yoo et al. [18], the authors contended that the ground-truth labels exhibit a significant correlation with the final performance.

### 2.2. KGs and LLMs

In general, the knowledge in KGs is called structured knowledge, while the knowledge stored in LLMs is known as parameterized knowledge. In practical applications, there is no strict upstream–downstream relationship between LLMs and KGs. For instance, pretrained Transformers can be employed for cross-domain KG completion [19,20]. The integration of LLMs and KGs can also take different forms, such as KG-enhanced LLMs or collaborative interaction. DeepKE-LL [21], originating from the fine-tuning of Large Language Model Meta AI (LLaMA) using low-rank adaptation (LoRA), parses data to accomplish knowledge extraction tasks. It also constructs an instruction-based fine-tuning dataset to enhance the capacity of LLMs for domain-specific knowledge extraction. KEPLER [22] adopts a shared Transformer-based encoder that incorporates a KG. K-BERT [23] introduces triplets into sentences using a visibility matrix to infuse LLMs with structured knowledge. The structured domain knowledge of the sentences is introduced in the embedding stage through the visibility matrix.

ERNIE [24] introduces a dual-stacked encoder to unify heterogeneous information from tokens and entities into a united space. KagNet [25] enhances input text representations by encoding KGs, thereby improving the capabilities of LLMs. REALM [26] employs knowledge retrieval to assist LLMs in acquiring knowledge from corpora during pretraining, resulting in notable performance enhancements in open-domain Q&A tasks.

As summarized above, many studies have explored enhancing the performance of LLMs using KGs from various perspectives. However, in specialized domains where higher accuracy and deep expertise are essential, general-purpose methods often fall short. These methods may misinterpret or oversimplify specialized terminology, fail to recognize uncommon relationships, or overlook critical domain-specific insights. Existing approaches often struggle to effectively integrate and apply the necessary prior knowledge, leading to suboptimal performance in tasks that require specialized expertise. Therefore, developing a more effective approach to integrating KGs with LLMs for domain-specific applications remains an area worthy of further exploration.

## 3. Methodology

**Figure 1:**
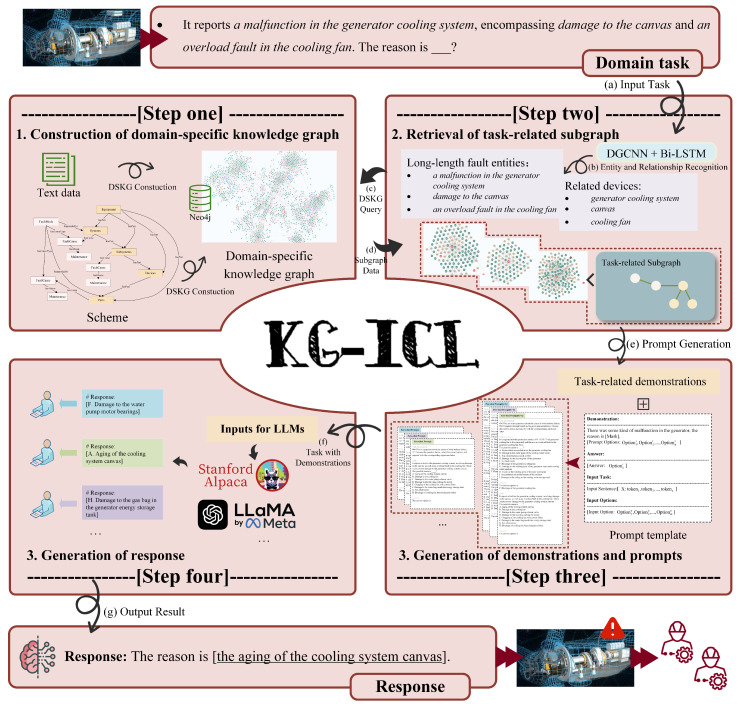
Process of KG-ICL. This process encompasses KG construction (Step one), relevant subgraph matching (Step two), and prompt generation (Step three). Ultimately, we utilize the generated prompts to assist LLMs in arriving at accurate conclusions for fault cause analysis (Step four).

In this section, we present the details of the proposed KG-ICL method for fault cause analysis [27]. The main flowchart is illustrated in Figure 1. We start by presenting the curation of a new dataset from collected fault records. Then, the design of KG-ICL and how it works are illustrated. Finally, we demonstrate how we reshape the task format into a multiple-choice format to streamline the evaluation process and efficiently apply constraints to the outputs of LLMs.

### 3.1. Dataset

To investigate the application of KG-ICL, we utilize diagnostic text data obtained from sensor networks. These records, which are made up of fault maintenance narratives provided by specialized personnel on site, provide a direct insight into the operational health of the equipment through textual descriptions derived from sensors. A comprehensive record includes four essential pieces of information: fault mode, cause of the fault, location of the fault, and maintenance strategy. To build an effective DSKG, the key entities and relationships within the field are identified.

The raw fault records comprise a total of 3200 fault texts. Through meticulous manual screening, we removed incomplete, inaccurate, and duplicated records. Ultimately, we refined the dataset to consist of 948 curated fault records. The dataset is divided into two different subsets, with no duplicate data:

- $D_{g}$: dataset for DSKG construction.
- $D_{t}$: dataset for testing.

The partitioning of these two datasets was carried out in a randomized manner, with a ratio of approximately 5:1. More precisely, $D_{g}$ encompasses 760 texts, while $D_{t}$ contains 188 texts. Using the test data set as an example, we performed a statistical analysis on the distribution of fault locations within the data set.

### 3.2. DSKG Construction

Here, we introduce the construction of the DSKG. We design a logically organized schema layer. In addition, we provide detailed information on the strategies used to retrieve subgraphs of interest from the DSKG and to generate task-related demonstrations. Finally, we outline how KG-ICL is employed to address domain-specific practical tasks.

#### 3.2.1. Schema Layer

A top-down approach is adopted to construct the DSKG. During the schema design process, domain-specific rules [28] and abstract concepts are integrated into a unified framework. We extract information, including fault modes, causes, locations, and maintenance strategies, from the well-organized $D_{g}$ dataset to help design the schema layer of the DSKG based on predefined rules [29]. The schema layer consists of two interacting sublayers: the equipment-related schema layer and the fault-related schema layer.

**Figure 2:**
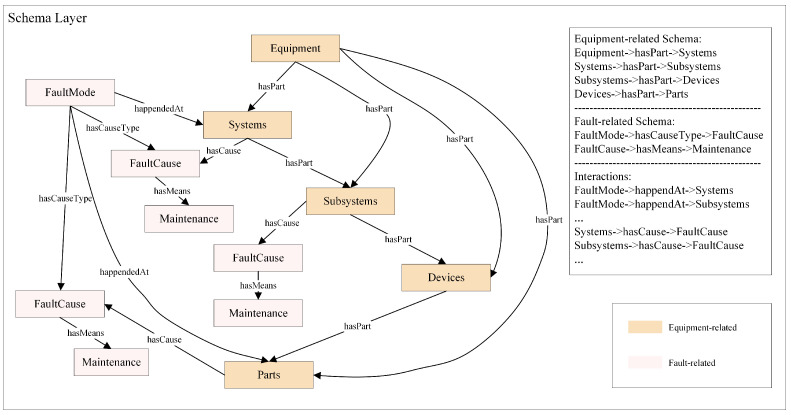
Schema layer of the DSKG. The schema layer is divided into two main parts: an equipment-related sublayer and a fault-related sublayer.

We design the equipment-related schema layer to account for the actual equipment structure of devices. This information is organized within the DSKG in a tree-like structure that mirrors the actual equipment hierarchy. The fault-related schema layer is composed of fault entities extracted from the dataset and interacts with the equipment-related schema layer [30,31], as shown in Figure 2.

#### 3.2.2. Construction of the DSKG

A KG is typically represented as a set of triples of the form ($e_{i},r_{k},e_{j}$), where $e_{i}$ and $e_{j}$ denote entities and $r_{k}$ denotes the relationship between them.

In dataset $D_{g}$, each text is represented as $X=\{x_{1},x_{2},x_{3},...,x_{n}\}$, where $x_{i}$ represents a token in the text. We extract information from the text regarding fault modes $e_{i}^{m}$, fault causes $e_{i}^{c}$, and maintenance strategies $e_{i}^{s}$.

(1) $$E=\left\{e_{i}^{m},e_{i}^{c},e_{i}^{s}\right\}=f_{NER}\left(X\right)$$

(2) $$R=\left\{r_{k}\right\}=f_{RE}\left(X\right)$$

Considering the existence of long-length entities in $D_{g}$, we employ a DGCNN to extract long-length entities and their relationships. The DGCNN architecture includes a gating mechanism and dilated convolution to achieve long-length entity extraction.

Gating mechanism:

(3) $$\begin{array}{cc} & Y=ConvD_{1}\left(X\right)⊗\sigma \left(ConvD_{2}\left(X\right)\right) \end{array}$$

Additive self-attention:

(4) $$x=Encoder\left(x_{1},x_{2},...,x_{n}\right)=\sum_{i=1}^{n}\lambda_{i}x_{i}$$

(5) $$\lambda_{i}=softmax\left(\alpha^{⊤}tanh\left(Wx_{i}\right)\right)$$

Here, α and *W* denote trainable parameters. The process of constructing the DSKG is summarized in Algorithm 1.
**Algorithm 1:** Process of Building DSKG**Input:**: Dataset $D_{g}$ **Output:**:
SPO list of entities and relationships
1:triplets = []2:**for** text $\in D_{g}$ **do**3:   // Tokenizing4:   text_embedding = Tokenizer(text)5:   // Parse the sentence to identify subject, predicate, and object6:   spos = DelitedGatedCNN(text_embedding)7:   **for** spo ∈ spos **do**8:     (subject, predicate, object) = spo9:     // Create a triplet and add it to the list10:     triplet = (subject, predicate, object)11:     triplets.append(triplet)12:   **end for**13:**end for**14:**return** triplets15://Build DSKG using pyneo and Neo4J16:DSKG_Construction(triplets)

**Figure 3:**
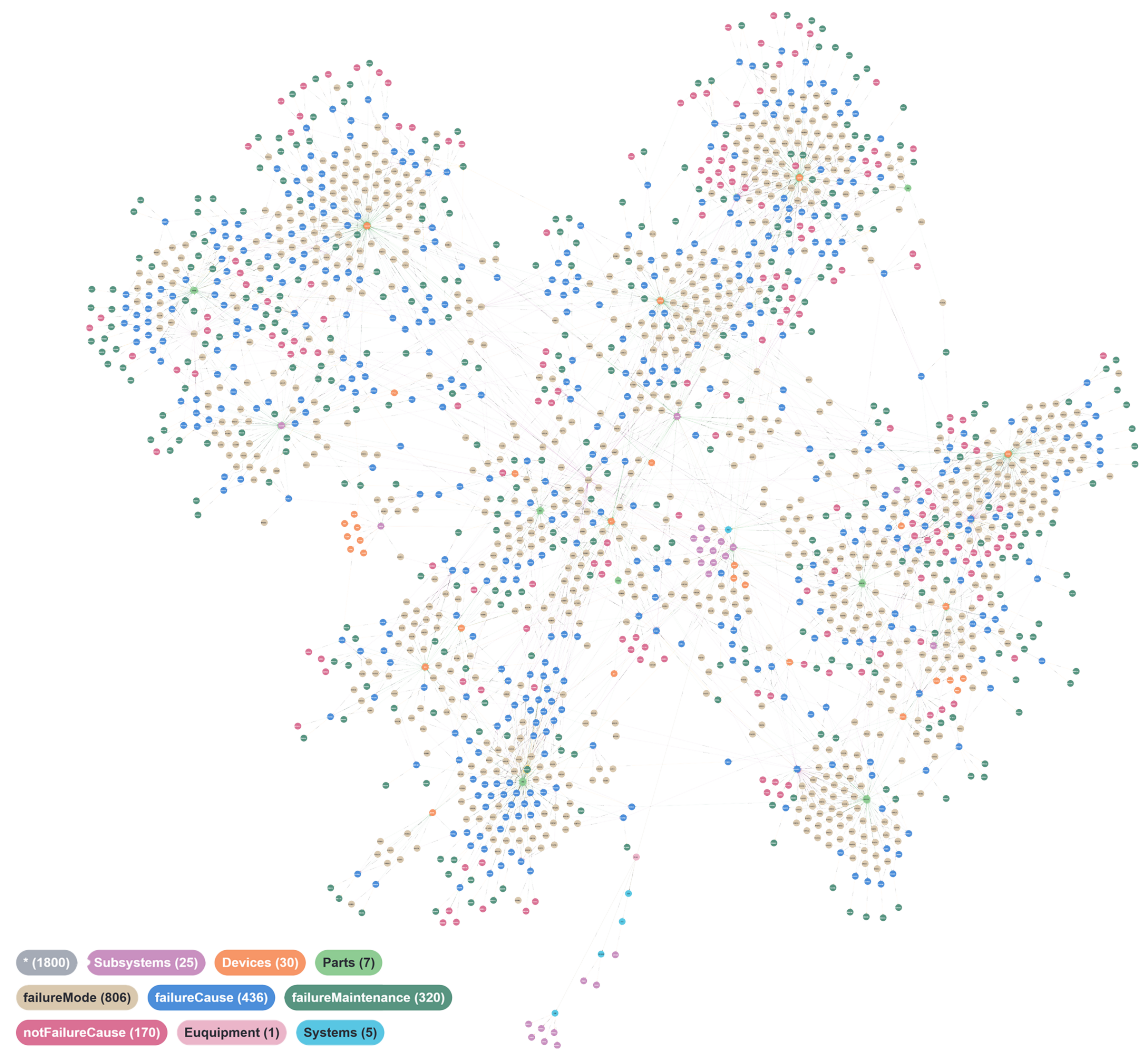
Part of a DSKG. Different colors in the figure represent different types of nodes, a total of 1800 nodes (*★*), the number of different nodes is also indicated in the legend.

We have already defined the attributes and relationships of nodes within the KG in the schema layer. After the extraction of entities and relationships, the schema layer is mapped onto the data layer. The data are stored in a Neo4J graph database, completing the construction of the DSKG. After the integration of the fault information from the dataset into a DSKG, the final graph comprises 1800 entities and 3353 relationships [32]. Figure 3 shows a visual representation of the DSKG.

### 3.3. Prompt Construction

After the construction of the DSKG, the next step involves refining the task workflow for practical applications. For each task in $D_{t}$, a relevant subgraph is retrieved from the DSKG. Furthermore, the task format is adjusted to simplify the evaluation process while maximizing the utilization of structured information from the DSKG.

#### 3.3.1. Subgraph Retrieval

**Figure 4:**
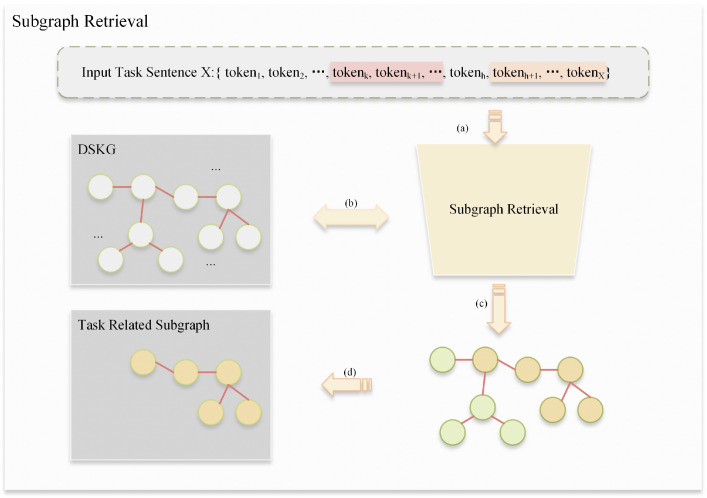
Subgraph retrieval. Subgraph retrieval is carried out mainly by means of a subgraph retriever. The process includes (**a**) data input, (**b**) KG query, (**c**) data filtering, and (**d**) subgraph generation. The white nodes represent entities within the knowledge graph, while the yellow and green nodes denote task-relevant nodes. Among these, the yellow nodes exhibit a higher degree of relevance to the current task.

For practical purposes, potentially relevant information must be extracted from the DSKG. To this end, we carefully choose the most pertinent parts of the graph through a subgraph matching process to obtain the subgraph of interest [33], as shown in Figure 4.

**Figure 5:**
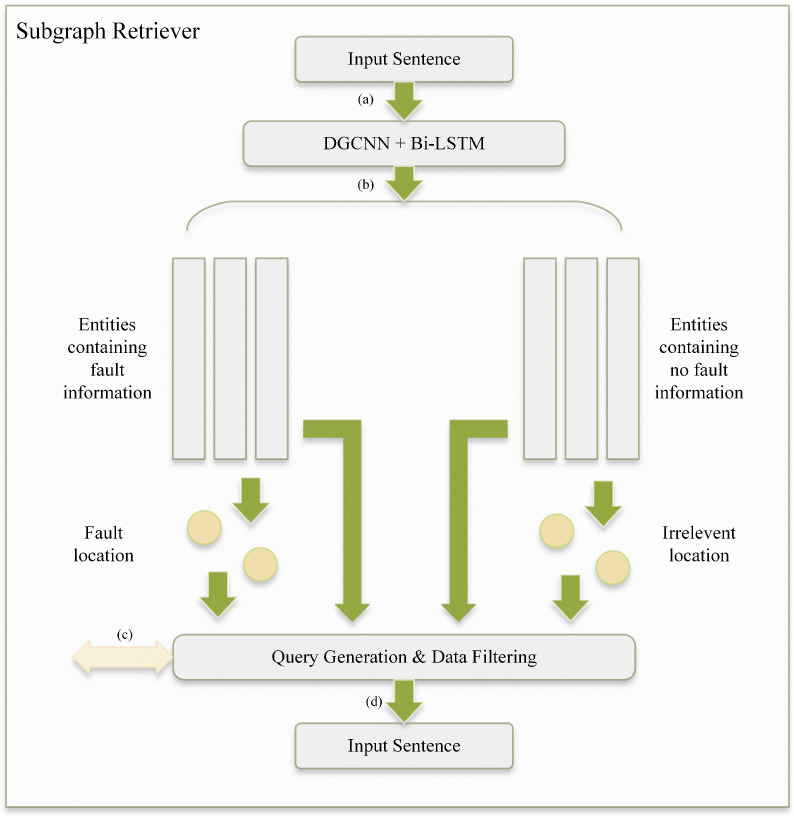
Subgraph retriever. (**a**) Coarse-grained classification is performed on the long-length entities extracted from the input text to determine whether they contain fault information. (**b**) Next, we perform word segmentation to obtain equipment-related information and (**c**) identify potentially relevant parts of the DSKG. (**d**) Finally, the relevant subgraph is obtained based on similarity to retrieve the information of interest.

The entities pertaining to the test task are extracted utilizing the DGCNN as previously discussed. Subsequently, BERT-BiLSTM is employed for coarse-grained classification of these entities to distinguish those that contain fault-related information. This allows us to obtain fault information about relevant equipment based on segmentation. Additional details regarding the subgraph retriever are shown in Figure 5.

For dealing with long-length entities in the DSKG, the long-length entity similarity (LES) metric is introduced to identify highly relevant matching results. Given a long-length entity phrase $q_{t}$ and a list of long-length entities to be matched, denoted by $Q=(q_{1},q_{2},...,q_{n})$, the similarity score between two entities is calculated as follows:

(6) $$\begin{array}{c} p_{i}=f\left(q_{t},q_{i}\right)=\varepsilon \times \zeta \left(q_{t},q_{i}\right)+\left(1-\varepsilon \right)\times \gamma \left(q_{t},q_{i}\right) \end{array}$$

Here, the parameter ε is adjustable. ζ(∗) represents the cosine similarity based on the sentence embeddings, and γ(∗) denotes the similarity in terms of the longest common subsequence (LCS):

(7) $$\begin{array}{cc} \; & \zeta \left(q_{t},q_{i}\right)=cos\left(SentenceEmbedding\left(q_{t},q_{i}\right)\right) \end{array}$$

(8) $$\begin{array}{cc} \; & \gamma \left(q_{t},q_{i}\right)=rough_{l}\left(q_{t},q_{i}\right) \end{array}$$

The cosine similarity is a commonly used metric, and the LCS similarity is calculated as follows:

(9) $$\begin{array}{c} rough_{l}\left(q_{t},q_{i}\right)=\frac{\left(1+\beta^{2}\right)\times \frac{\xi^{2}}{m\times n}}{\frac{\xi }{m}+\beta^{2}\times \frac{\xi }{n}} \end{array}$$

Here, ξ represents the length of the longest common subsequence. *m* represents the length of $q_{t}$, and *n* is the length of $q_{i}$. In our experiments, the parameter β is set to 1.

#### 3.3.2. Prompt Construction

For the task text $t_{o}$, we organize the retrieved subgraph into *k* relevant demonstrations $\psi (T_{i})$, which have a format similar to that of the input text.

(10) $$\begin{array}{c} \psi \left(T_{i}\right):\left(t_{1},N_{1}\right),\left(t_{2},N_{2}\right),\cdots ,\left(t_{k},N_{k}\right) \end{array}$$

In each demonstration, *N* is a set containing the correct answer. Similarly, for the input text, the subgraph is utilized to generate the corresponding answers $N_{t_{o}}$ [34]. Given the task-related demonstrations, we expect LLMs to make the correct selection and return the index of the best answer $n_{t_{o}}$ [35,36,37].

(11) $$\begin{array}{c} n_{t_{o}}=argmax_{n\in N_{t_{o}}}P\left(n|t_{1},N_{1},t_{2},N_{2},\cdots ,t_{k},N_{k},t_{o}\right) \end{array}$$

**Figure 6:**
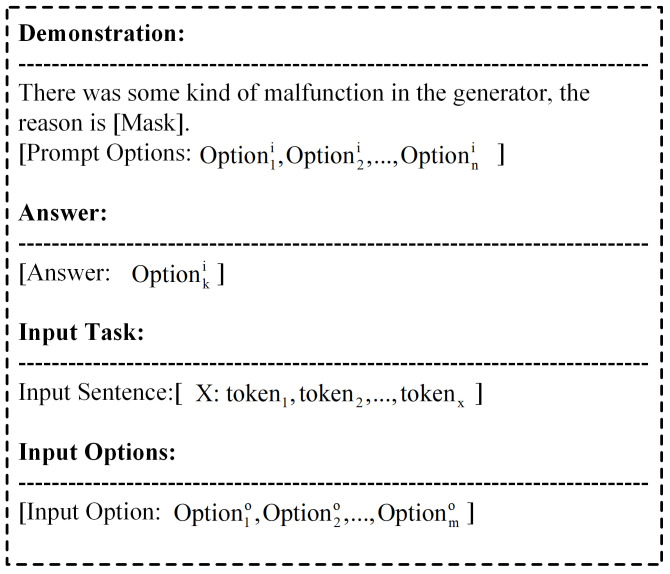
Prompt template.

The input format for prompts with demonstrations is shown in Figure 6.

## 4. Experiments

### 4.1. Experimental Setting

To validate the effectiveness of the KG-ICL method, we design a series of experiments involving LLMs of various sizes and different types of tasks. We compare the performance of task prompts generated using the KG-ICL method against traditional random prompts. The experimental results demonstrate the potential of the KG-ICL method to enhance the causal analysis capabilities of LLMs in the field of equipment diagnostics. In the subgraph retrieval process, the parameter ε is set to 0.6. Regarding the demonstrations, there are k=10 demonstrations corresponding to each text in the test dataset.

In these experiments, demonstrations are generated using the DSKG. We use the random domain-related demonstrations as an alternative to conventional demonstrations for experimentation. Demonstrations that are related to the given task are referred to as task-related demonstrations. Three types of experiments are conducted: no demonstrations, domain-related demonstrations, and task-related demonstrations.

No demonstrations. All demonstrations are removed to evaluate the performance of the LLMs when no demonstration data are available.

Domain-related demonstrations. Random demonstrations are used as demonstrations for tasks, and the scale of the demonstrations for each task is the same as in KG-ICL. This experiment serves as a control experiment representing common methods without the use of KGs.

Task-related demonstrations. Task-related demonstrations are generated through the KG-ICL method, which are highly relevant to the specific task. This experiment is conducted to evaluate the proposed KG-ICL method.

Considering the available hardware resources at our disposal, we choose a selection of commonly used LLMs for use in these experiments. To comprehensively evaluate the performance of the proposed KG-ICL method, we include LLMs with parameter scales ranging from 700 M to 1.8 T.

Our aim is to illustrate the effectiveness of KG-ICL. Considering differences in architecture and training corpora, we compare several LLMs, noting their versions, training strategies, and parameter scales for fair evaluation.

We choose several Chinese LLaMA models, a Chinese Vicuna model that was fine-tuned based on LLaMA, and Chinese Alpaca models that were further fine-tuned based on LLaMA with instructions specific to our experiments. In this series of models, 20k words are added to the original LLaMA dictionary, enhancing the model’s ability to process and generate.

We also report comparative experiments conducted with the highly acclaimed GPT-4 as well as similar models such as GPT-3.5 and GPT-2-Large [38,39].

The selected LLMs and their parameter scales are listed in Table 1.

### 4.2. Evaluation

For the evaluation of the results, we rely on manual assessments performed by domain experts to determine the accuracy of the outputs. Before presenting the evaluation results, we first establish a standardized and clear set of criteria to judge the outputs of the LLMs.

E: Error. The result is entirely incorrect, including both the identification of the fault cause and the identification of the fault location.

P: Position Correct. The analysis correctly identifies the fault location, but the analysis of the fault cause is incorrect.

C: Cause Correct. The result is correct, including both the analysis of the fault cause and the identification of the fault location.

Accuracy of Position Correct:

(12) $$\begin{array}{cc} & ACC_{PositionCorrect}=\frac{P+C}{P+C+E} \end{array}$$

Accuracy of Cause Correct:

(13) $$\begin{array}{cc} & ACC_{CauseCorrect}=\frac{C}{P+C+E} \end{array}$$

### 4.3. Results

The results of experiments based on different values of k are presented in Table 2. The LLMs were tested under conditions of k = 1, 3, 5, 8, and 10 [40] to assess the model performance with varying numbers of demonstrations. We compare the standard ICL method (domain-related demonstrations) with the task-related demonstrations generated by our KG-ICL method to examine the impacts of the different types of demonstrations on model performance.

**Figure 7:**
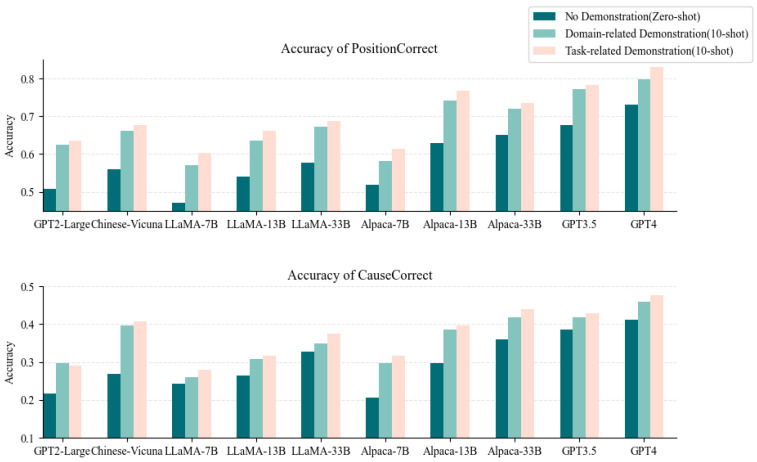
Accuracy in terms of Position Correct and Cause Correct outcomes (k = 10).

The results of experiments when k is set to 10 are presented in Table 3 and Figure 7, encompassing different LLMs and three variations of ICL.

**Figure 8:**
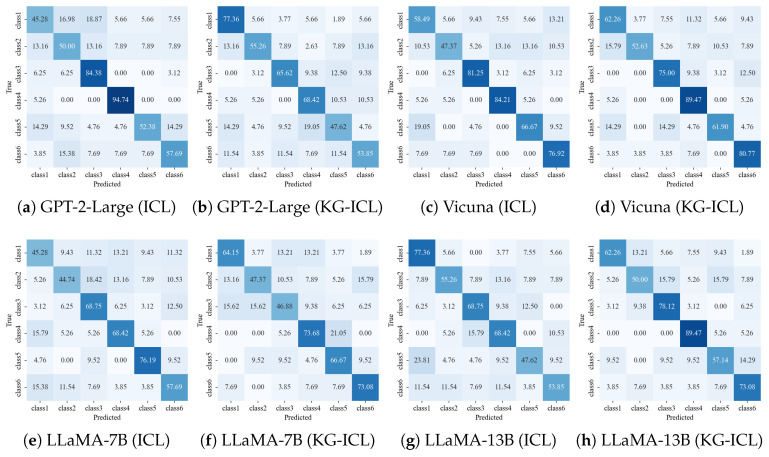

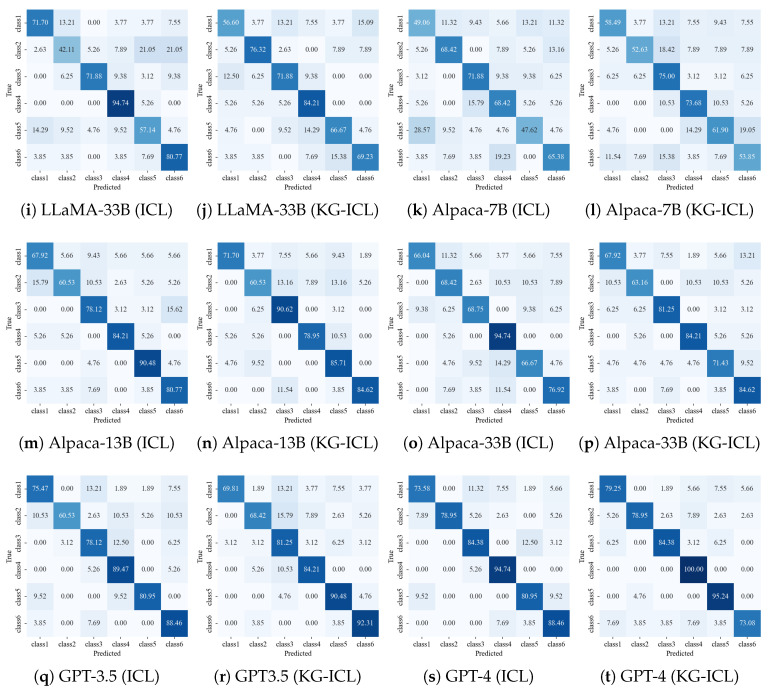
The confusion matrix of the results (k = 10). A more intense blue color signifies a higher proportion.

To present the experimental results in a clear way, the confusion matrix derived from the results [41] is illustrated in Figure 8. This confusion matrix reflects the results of the experiments when the parameter k is set to 10.

## 5. Discussion

The results in Table 2 show an improvement in the model performance with an increase in the number of demonstrations. This indicates a positive correlation between model performance and the quantity of demonstrations provided. Additionally, under the same number of demonstrations, the models perform better when the KG-ICL method is introduced. Next, to compare the enhancement in model performance enabled by KG-ICL, we specifically analyze and compare our results based on the condition of k = 10.

As shown in Table 3, there is a consistent increase in precision with an increasing number of model parameters, which is in line with our expectations. However, our primary focus lies in understanding how LLMs perform under different variations in the ICL. Thus, we wish to assess the extent to which our proposed KG-ICL method enhances the performance of LLMs in domain-specific applications.

Table 4 presents the performance of the LLMs under the three different ICL variations—no demonstrations, domain-related demonstrations, and task-related demonstrations—based on the Position Correct criterion. Table 4 shows that under the Position Correct criterion, the performance of LLMs with domain-related demonstrations is 1.0–11.6% better than the performance with no demonstrations. Consequently, under the proposed KG-ICL method, the performance of the LLMs given tasks-related demonstrations is higher than that of domain-related demonstrations, with enhancements ranging from 1.1% to 6.9%.

Similarly, Table 5 presents the performance of the LLMs based on the Cause Correct criterion. In Table 5, we can again observe that under the Cause Correct criterion, the performance of the LLMs with domain-related demonstrations is 2.1–12.7% better than with no demonstrations. Consequently, under the proposed KG-ICL method, the performance of the LLMs given tasks-related demonstrations is higher than that of domain-related demonstrations, with enhancements ranging from 1.0% to 2.7%. In particular, for GPT-2-Large, there is almost no discernible difference in performance between the conditions of no demonstrations and task-related demonstrations. We attribute this phenomenon to the relatively small parameter scale of this model, which limits the extent to which higher-quality demonstrations can enhance its performance.

In the specialized domain, the LLMs consistently exhibit good performance. Employing a DSKG for prompt generation significantly streamlines the process of prompt design. These experiments demonstrate that the proposed KG-ICL method excels not only in delivering demonstrations of the same format but also in generating prompts that are well aligned with the specific task at hand. Through the utilization of KG-ICL to construct demonstrations and prompts, a cooperative link between the structured knowledge stored in a KG and the parameterized knowledge of LLMs can be established for domain-specific applications.

### 5.1. Cross-Validation Experiments

To further validate the robustness of our KG-ICL method, we have incorporated cross-validation experiments. These experiments involve partitioning the dataset into four different subsets, with each subset being utilized as a test set in turn, while the others are employed for DSKG construction.

Experiments are executed using the Chinese-Alpaca-33B, with four different test datasets drawn at random without repetition. These experiments consistently align with our previous results, as detailed in Table 6.

### 5.2. Ablation Study on the LES Component

The proposed LES component plays an important role in the KG-ICL method proposed in this study, aiming to enhance KG retrieval for specialized-domain texts by considering the similarity between long-length domain entities. To validate the effectiveness and necessity of the LES calculation, a series of ablation experiments were conducted.

We designed a comparative experiment by removing the LES component from the KG-ICL method and instead using the similarity of sentence embedding. In this scenario, the model fails to fully capture the semantic relationships between entities, potentially leading to suboptimal performance in text analysis.

Subsequently, we conducted comparisons across multiple LLMs, comparing the performance of the KG-ICL method with and without the inclusion of the LES component, as shown in Table 7. The introduction of the LES significantly improves the accuracy and relevance of KG retrieval, especially when dealing with entities from a specialized domain, achieving enhanced effects compared to traditional methods.

These ablation experiment results confirm the indispensability of the LES component in the KG-ICL method, demonstrating its effectiveness for text analysis.

## 6. Conclusions

This paper presents an innovative KG-ICL method, designed to enhance the causal analysis capabilities of LLMs for processing texts within the equipment diagnostics domain. By constructing a DSKG and integrating an LES, our approach provides LLMs with structured knowledge, significantly improving their accuracy and efficiency in equipment diagnostics. The performances of nine different LLMs are compared in experiments employing three distinct types of ICL demonstrations.

Our experimental results demonstrate that, compared to traditional ICL methods, the KG-ICL method consistently outperforms across various tasks, confirming its effectiveness and feasibility in practical applications.

Our study offers some helpful insights regarding the use of KGs and LLMs in domain-specific applications:

- Enriching prompt templates with KGs yields superior results in terms of demonstration quality compared to manual approaches.
- The integration of KGs when generating prompts leads to more task-relevant prompts.
- The integration of structured knowledge from KGs into prompts enhances the accuracy of the content generated by LLMs.

In conclusion, our research presents an approach that facilitates the integration of KGs and LLMs. Through this method, we have successfully demonstrated the application of KG-enhanced prompts for LLMs in specialized domains, achieving satisfactory results in equipment diagnostics. The KG-ICL method introduces a new technological tool for text analysis within this domain, providing an innovative solution for equipment maintenance. Furthermore, the modular and flexible design of this method ensures its high scalability and adaptability, enabling it to address the ever-evolving challenges in equipment diagnostics. 

## Figures and Tables

**Table 1:** The LLMs utilized in the experiments, including GPT-2-Large, Chinese Vicuna, Chinese LLaMA, Chinese Alpaca, GPT-3.5, GPT-4, and the size of their parameters.

Model	Params	Model	Params
GPT-2-Large	774 M	Chinese-Alpaca-pro	7 B
Chinese-Vicuna	7 B	Chinese-Alpaca-pro	13 B
Chinese-LLaMA-plus	7 B	Chinese-Alpaca-pro	33 B
Chinese-LLaMA-plus	13 B	ChatGPT (GPT-3.5)	175 B
Chinese-LLaMA-plus	33 B	GPT-4	1.8 T

**Table 2:** Test results with different numbers of demonstrations *k*.

Model	k	Method	${\mathrm{ACC}}_{\mathrm{Cause}\mathrm{Correct}}$	${\mathrm{ACC}}_{\mathrm{Position}\mathrm{Correct}}$	Method	${\mathrm{ACC}}_{\mathrm{Cause}\mathrm{Correct}}$	${\mathrm{ACC}}_{\mathrm{Position}\mathrm{Correct}}$
	1-shot		0.307	0.529		0.280	0.540
	3-shot		0.275	0.556		0.270	0.561
GPT-2-Large	5-shot	ICL	0.270	0.571	KG-ICL	0.259	0.582
	8-shot		0.275	0.603		0.270	0.614
	10-shot		0.296	0.624		0.291	0.635
	1-shot		0.286	0.582		0.286	0.593
	3-shot		0.312	0.603		0.296	0.614
Chinese-Vicuna	5-shot	ICL	0.360	0.630	KG-ICL	0.413	0.646
	8-shot		0.413	0.646		0.354	0.651
	10-shot		0.397	0.661		0.407	0.677
	1-shot		0.228	0.503		0.254	0.508
	3-shot		0.228	0.540		0.259	0.545
Chinese-LLaMA-7B	5-shot	ICL	0.275	0.545	KG-ICL	0.243	0.550
	8-shot		0.212	0.561		0.243	0.571
	10-shot		0.259	0.571		0.280	0.603
	1-shot		0.291	0.577		0.286	0.582
	3-shot		0.296	0.587		0.275	0.593
Chinese-LLaMA-13B	5-shot	ICL	0.254	0.603	KG-ICL	0.270	0.614
	8-shot		0.302	0.614		0.333	0.624
	10-shot		0.307	0.635		0.317	0.661
	1-shot		0.312	0.582		0.270	0.603
	3-shot		0.296	0.619		0.280	0.630
Chinese-LLaMA-33B	5-shot	ICL	0.333	0.640	KG-ICL	0.296	0.656
	8-shot		0.360	0.667		0.333	0.656
	10-shot		0.349	0.672		0.376	0.688
	1-shot		0.238	0.534		0.243	0.540
	3-shot		0.275	0.550		0.280	0.556
Chinese-Alpaca-7B	5-shot	ICL	0.243	0.550	KG-ICL	0.238	0.566
	8-shot		0.275	0.550		0.286	0.582
	10-shot		0.296	0.582		0.317	0.614
	1-shot		0.323	0.667		0.418	0.677
	3-shot		0.349	0.698		0.354	0.704
Chinese-Alpaca-13B	5-shot	ICL	0.397	0.709	KG-ICL	0.339	0.720
	8-shot		0.360	0.720		0.370	0.725
	10-shot		0.386	0.741		0.397	0.767
	1-shot		0.360	0.661		0.370	0.667
	3-shot		0.354	0.683		0.418	0.688
Chinese-Alpaca-33B	5-shot	ICL	0.365	0.693	KG-ICL	0.365	0.704
	8-shot		0.402	0.714		0.392	0.720
	10-shot		0.418	0.720		0.439	0.735
	1-shot		0.386	0.693		0.402	0.720
	3-shot		0.354	0.725		0.429	0.730
ChatGPT-3.5	5-shot	ICL	0.365	0.741	KG-ICL	0.429	0.746
	8-shot		0.418	0.746		0.413	0.757
	10-shot		0.418	0.772		0.429	0.783
	1-shot		0.455	0.741		0.423	0.751
	3-shot		0.429	0.772		0.450	0.767
GPT-4	5-shot	ICL	0.450	0.772	KG-ICL	0.434	0.783
	8-shot		0.466	0.794		0.476	0.794
	10-shot		0.460	0.799		0.476	0.831

**Table 3:** Results with no demonstrations, domain-related demonstrations (ICL), and task-related demonstrations (KG-ICL) (10-shot prompts).

Accuracy	Method	GPT-2-Large	Chinese-Vicuna	LLaMA-7B
$ACC_{CauseCorrect}$	No demonstrations	0.254	0.296	0.212
	ICL	0.249	0.360	0.243
	KG-ICL	0.270	0.397	0.275
$ACC_{PositionCorrect}$	No demonstrations	0.513	0.614	0.534
	ICL	0.556	0.630	0.571
	KG-ICL	0.577	0.661	0.635
**Accuracy**	**Method**	**LLaMA-13B**	**LLaMA-33B**	**Alpaca-7B**
$ACC_{CauseCorrect}$	No demonstrations	0.148	0.328	0.249
	ICL	0.238	0.349	0.259
	KG-ICL	0.265	0.376	0.270
$ACC_{PositionCorrect}$	No demonstrations	0.593	0.577	0.656
	ICL	0.640	0.672	0.667
	KG-ICL	0.667	0.688	0.683
**Accuracy**	**Method**	**Alpaca-13B**	**Alpaca-33B**	**GPT-3.5**
$ACC_{CauseCorrect}$	No demonstrations	0.296	0.360	0.386
	ICL	0.365	0.370	0.365
	KG-ICL	0.397	0.392	0.413
$ACC_{PositionCorrect}$	No demonstrations	0.630	0.651	0.677
	ICL	0.698	0.667	0.741
	KG-ICL	0.709	0.709	0.757
**Accuracy**	**Method**	**GPT-4**		
$ACC_{CauseCorrect}$	No demonstrations	0.413		
	ICL	0.460		
	KG-ICL	0.476		
$ACC_{PositionCorrect}$	No demonstrations	0.730		
	ICL	0.799		
	KG-ICL	0.831		

**Table 4:** Results in terms of the Position Correct criterion (10-shot prompts). ↑ indicates the percentage of the result improvement, and the ↓ indicates the opposite.

Models	Domain-Related vs. No (%)	Task-Related (KG-ICL) vs. Domain-Related
GPT-2-Large	↑ 11.6	↑1.1
Chinese-Vicuna	↑1.0	↑1.6
Chinese-LLaMA-7B	↑1.0	↑3.2
Chinese-LLaMA-13B	↑9.5	↑2.6
Chinese-LLaMA-33B	↑9.5	↑1.6
Chinese-Alpaca-7B	↑6.3	↑3.2
Chinese-Alpaca-13B	↑11.1	↑2.6
Chinese-Alpaca-33B	↑6.9	↑6.9
GPT-3.5	↑9.5	↑1.1
GPT-4	↑6.9	↑3.2

**Table 5:** Results in terms of the Cause Correct criterion (10-shot prompts). ↑ indicates the percentage of the result improvement, and the ↓ indicates the opposite.

Models	Domain-Related vs. No (%)	Task-Related (KG-ICL) vs. Domain-Related
GPT-2-Large	↑7.9	↓0.5
Chinese-Vicuna	↑12.7	↑1.0
Chinese-LLaMA-7B	↑1.6	↑2.1
Chinese-LLaMA-13B	↑4.2	↑1.0
Chinese-LLaMA-33B	↑2.1	↑2.7
Chinese-Alpaca-7B	↑9.0	↑2.1
Chinese-Alpaca-13B	↑9.0	↑1.1
Chinese-Alpaca-33B	↑5.8	↑2.1
GPT-3.5	↑3.2	↑1.1
GPT-4	↑4.7	↑1.6

**Table 6:** Cross-validation experiments.

Dataset	ACC_*Cause Correct*_	ACC_*Position Correct*_
D_g, D_t	0.439	0.735
D_g1, D_t1	0.444	0.741
D_g2, D_t2	0.434	0.730
D_g3, D_t3	0.444	0.725

**Table 7:** Ablation experiments on the LES component.

Model	1-Shot		3-Shot		5-Shot		8-Shot		10-Shot	
	KG-ICL	KG-ICL (LES)	KG-ICL	KG-ICL (LES)	KG-ICL	KG-ICL (LES)	KG-ICL	KG-ICL (LES)	KG-ICL	KG-ICL (LES)
GPT-2-Large	0.513	0.540	0.556	0.561	0.561	0.582	0.587	0.614	0.603	0.635
Chinese-Vicuna	0.571	0.593	0.603	0.614	0.624	0.646	0.646	0.651	0.661	0.677
LLaMa-7B	0.492	0.508	0.503	0.545	0.534	0.550	0.556	0.571	0.566	0.603
LLaMa-13B	0.556	0.582	0.577	0.593	0.593	0.614	0.603	0.624	0.640	0.661
LLaMa-33B	0.571	0.603	0.624	0.630	0.640	0.656	0.651	0.656	0.677	0.688
Alpaca-7B	0.524	0.540	0.550	0.556	0.556	0.566	0.577	0.582	0.608	0.614
Alpaca-13B	0.640	0.667	0.693	0.704	0.704	0.720	0.709	0.725	0.741	0.767
Alpaca-33B	0.651	0.667	0.677	0.688	0.683	0.704	0.709	0.720	0.714	0.735
GPT-3.5	0.693	0.720	0.725	0.730	0.741	0.746	0.751	0.757	0.767	0.783
GPT-4	0.735	0.751	0.757	0.767	0.772	0.783	0.794	0.794	0.815	0.831

## Data Availability

Data are available upon reasonable request.
